# Targeted Drug Delivery Systems for the Treatment of Glaucoma: Most Advanced Systems Review

**DOI:** 10.3390/polym11111742

**Published:** 2019-10-24

**Authors:** Olga Cegielska, Paweł Sajkiewicz

**Affiliations:** Laboratory of Polymers and Biomaterials, Institute of Fundamental Technological Research Polish Academy of Sciences, Pawińskiego 5b, 02-106 Warsaw, Poland; psajk@ippt.pan.pl

**Keywords:** hydrogels, nanofibers, electrospinning, glaucoma, ophthalmology

## Abstract

Each year, new glaucoma drug delivery systems are developed. Due to the chronic nature of the disease, it requires the inconvenient daily administration of medications. As a result of their elution from the eye surface and penetration to the bloodstream through undesired permeation routes, the bioavailability of active compounds is low, and systemic side effects occur. Despite numerous publications on glaucoma drug carriers of controlled drug release kinetics, only part of them consider drug permeation routes and, thus, carriers’ location, as an important factor affecting drug delivery. In this paper, we try to demonstrate the importance of the delivery proximal to glaucoma drug targets. The targeted delivery can significantly improve drug bioavailability, reduce side effects, and increase patients’ compliance compared to both commercial and scientifically developed formulations that can spread over the eye surface or stay in contact with conjunctival sac. We present a selection of glaucoma drug carriers intended to be placed on cornea or injected into the aqueous humor and that have been made by advanced materials using hi-tech forming methods, allowing for effective and convenient sustained antiglaucoma drug delivery.

## 1. Introduction

The human eye is a complex organ composed of many specialized elements of various structure and properties, combining their functions for a common goal—eyesight. As a result of the eye’s multicomponent structure, it can be susceptible to various diseases. The internal part of the eye is, however, well protected from undesirable molecules by its external parts, possessing barrier, antimicrobial, and antibacterial properties [1]. At the same time, these parts constitute a main route for ophthalmic drugs. The consequence of such duality is a debate regarding the permeability of the eye [1,2,3,4,5].

Open-angle glaucoma is an eye disease leading to gradual loss of sight. Pharmaceutical and surgical treatment, concentrating on the management of intraocular pressure (IOP), can significantly slow the adverse changes and prevent from blindness [6]. Surgical treatments include laser treatment and implantation of devices, lowering IOP via an outflow mechanism (MIGS) [7]. However, not every patient can or want to undergo a surgery, which is why pharmaceutical treatment is most common.

Due to the nature of topical formulations, their elution from the eye surface, as well as penetration to the bloodstream through the eye’s vessels and nasolacrimal duct, is easy, resulting in low drug bioavailability [8,9,10]. For most antiglaucoma drugs multiple daily dosing is obligatory to maintain the drug in the therapeutic window. There is a risk of side effects, as all of the antiglaucoma active ingredients manifest systemic action [11,12]. Additionally, administration of almost all glaucoma drugs results in eye irritation. The route of drug absorption is therefore relevant.

It is therefore clear that novel glaucoma drug delivery systems are needed. To significantly improve drug bioavailability, reduce side effects, and increase patient compliance, their action spot should be narrowed when compared to traditional topical formulations. For this purpose, a deeper understanding of transport rates across ocular tissues is crucial. The literature analysis has led to the conclusion that the most preferable localization of novel carriers is in the closest area of the drug targets responding to a glaucoma drug. In this paper, a selection of the most interesting and innovative glaucoma drug carriers are presented.

## 2. Open-Angle Glaucoma

### 2.1. Basics and Prognosis

Glaucoma refers to a group of optic neuropathies that share a common consequence in the progressive degeneration of retinal ganglion cells with their axons, as well as the supporting glia and vasculature [13]. In most glaucoma types, the impairment of the nerve system is a result of the imbalance between aqueous humor production and drainage, causing a pressure on the optic nerve. It is manifested by an elevated IOP and can lead to blindness, which cannot be restored [14]. However, it is preventable and further damage can be minimized with appropriate treatment, which focus on the management of IOP [6].

The prevalence and unfavorable prognosis of untreated glaucoma qualifies it for social civilization diseases. According to statistics, glaucoma affects about 4% of people aged 40–80 years worldwide [15]. It is estimated that about 70 million people worldwide have glaucoma, from which approximately 10% are bilaterally blind [16].

Primary open-angle glaucoma is the most common form of glaucoma. Contrary to angle-closure glaucoma, the angle between the iris and cornea is wide and does not constitute an obstacle for the aqueous flow [17]. Clinical trials provide convincing evidence that lowering IOP prevents progression at both early and late stages of the disease and the degree of protection is proportional to the decrease in its value [6].

### 2.2. Antiglaucoma Drugs

There are various types of glaucoma drugs lowering IOP via different mechanisms—by increasing the uveoscleral outflow (prostaglandins), inhibiting the aqueous humor inflow (carbonic anhydrase inhibitors, β-blockers) or, in chronic treatment, both (α -blockers), as well as by increasing the trabecular meshwork outflow (cholinergic agonists). Administration of all of these is associated with ocular adverse effects while, in conjunction with other systemic diseases, it may cause or exacerbate systemic side effects [11,12].

Antiglaucoma drugs are salts of hydrophobic compounds. Most of them ionize partially or completely in the tear film at pH 7.6. Locations of their targets differ, but the ciliary body is common to all [18,19,20,21,22,23].

## 3. Traditional Glaucoma Drug Forms and Mechanisms of Their Delivery—An Overview

Drug delivery of ophthalmic drugs is mainly provided topically. The local delivery is facile as the eye is an easily accessible external organ. Furthermore, systemic delivery is less effective because of the blood–retinal barrier and the size ratio of the eye to the entire body [24]. Though the most commonly used topical ophthalmic drug forms are aqueous solutions, they are also available as oil-based vehicles and aqueous suspensions.

The pH of the formulation can be adjusted, either to enhance the drug’s solubility, or to increase the rate of un-ionized drug compounds, since it favors corneal penetration. This is achieved by the addition of eye-irritating pH modifiers [25]. Sometimes, acidic or alkaline storage is essential to prevent the drug from degradation [24]. The ocular irritation that it causes may exceed the elution effect as well as induce changes in the intraocular pressure and promote the dry eye disease [26]. Preservatives have also been reported to have a negative effect on the eye surface, including lachrymal film dysfunction, ocular hyperemia, dotted keratitis, or toxic keratopathy [27]. Elimination or replacement of these unfavorable ingredients from ophthalmic drug carriers is therefore highly desired. Despite how easy the topical drug administration to the eye appears to be, the eye’s anatomy, physiology, and biochemistry is designed to make it nearly impervious to foreign agents, which significantly impedes effective drug delivery [28]. The bioavailability of most active substances from commercially available formulations does not exceed 5% [8,9,10], and less than 3% of an applied dose reaches the aqueous humor. It is the most significant problem of ophthalmic drug delivery, and the reason for the use of high drug concentrations in conventional formulations. The reason lies mainly in the dual properties of eye structures, combining permeation and barrier properties. The cornea is a nonvascularized semipermeable structure, where the epithelium hinders the permeation of hydrophilic drugs and macromolecules, the stroma prevents the diffusion of highly lipophilic compounds that cross the epithelium, and the endothelium is a leaky lipophilic barrier. Sclera’s permeability is similar to corneal. While they are both a direct route for drug absorption to the ciliary body, conjunctiva—covering the latter—redirect most of the molecules to general circulation via the conjunctival sac [29,30,31,32].

In the case of liquid drug forms, a drug’s entry limitation into the anterior segment is reinforced by dynamic barriers. Formulations spread over the eye and become partially eluted from the eyeball with the tear film. This so-called precorneal drug loss is caused by the corneal reflex and tearing following drug administration. These factors, together with static permeation barriers, gravitation, and eyelid motion, redirect the drug to the conjunctival sack and nasolacrimal duct. It is estimated that through the latter, between 80% and 90% of the droplet applied in form of aqueous solutions drains [3]. Additionally, to negatively affecting the optical therapy effectiveness, it may cause side effects in the case of drugs of systemic action [11,12]. The permeation of the active substances from nonviscous water formulations is immediate and, therefore, the active substance stay in the therapeutic window is very short [33]. Also, for most ophthalmic drugs, multiple daily dosing is obligatory. It causes poor compliance, especially in elderly patients, and a risk of microbiological contamination, favored by improper drug instillation [34,35].

Oil-based vehicles and aqueous solutions of increased viscosity reduce the problem of drug elution because of a more stable attachment to the eye surface. The drug release is prolonged, and so is their stay in the therapeutic window; however, they all cause blurred vision. There are also commercially available drug-releasing eye implants for implantation in the conjunctival sac, the so-called inserts. The drug release from such structures is much longer compared to abovementioned formulations, as the insert slowly degrades in the location place, gradually freeing the drug into the environment. The ocular residence of the drug is therefore increased; however, the place of administration is highly improper and favors penetration into the bloodstream [36]. Disadvantages are also reported, including a foreign body sensation [37], a risk of unnoticed expulsion from the eye, or blurred vision in case of fast dissolving inserts [38].

From the available drug delivery methods, there are also invasive methods—drug injections. The drug is administered intravitreally and omits the blood–ocular barriers, allowing for obtaining drug levels higher than achievable by systemic or topical administration. It also allows for avoiding systemic side effects [4]. However, ocular side effects of repeated injections may occur, including development or intensification of the dry eye syndrome and inflammation of the interior of the eye [39,40]. It was also found that intravitreal dexamethasone implants can induce secondary glaucoma [41]. One obvious negative aspect of such a procedure is an unpleasant sensation during intervention.

As can be seen, although some improvements have been made in the field of antiglaucoma drug delivery, there is still no widespread alternative for aqueous solutions. This has prompted researchers to come up with more sophisticated solutions.

## 4. Modern Ophthalmic Drug Release Systems

### 4.1. General Description, Goals, and Challenges

Modern drug release systems are directed toward technologies and materials, allowing for a local, sustained, and prolonged delivery of therapeutic agents [42]. Narrowing the area of the delivery allows for reaching the drug target directly by the therapeutic agent. The constancy of the drug release and, therefore, of the drug delivery to the drug target prolong its stay in the therapeutic window, thus causing a significant increase in therapy effectiveness and the reduction of side effects from certain medications. It also allows for reduction of the drug dosage, which has a great economic importance and results in a major increase in drug compliance.

In general, drug carriers should be able to encapsulate the high concentration of a drug and provide a sustained drug release with minimum burst. Ophthalmic drug carriers should bring an additional advantage of eliminating ocular drawbacks of traditional drug forms, such as eye irritation, blurred vision, potency of infection, etc.

The development of materials science provides plenty of advanced biocompatible polymers with tunable mechanical properties and degradation rates able to serve as drug carriers [43]. They provide a protection for the drug, increasing its stability, and slow down the drug delivery. Most polymers used in drug delivery systems are biodegradable, with the biodegradation rate being a mechanism controlling drug release. Among them, there are stimuli-responsive polymers sensitive to pH, mechanical stimulation, light, or other factors, capable of releasing the drug at the proper site and amount only when triggered, achieving so-called intelligent delivery [44]. Their potential in ophthalmic applications have been shown in a visible light-controlled multicomponent bevacizumab delivery system. The drug release was effectively controlled by the adjustment of component concentrations, light intensity, and irradiation time [45]. No similar approaches for glaucoma treatment were made.

The predominance of vascular permeation, being the main reason for low drug bioavailability, indicates the need for carriers with a narrow, specific area of action, with the slightest contact with blood vessels. Therefore, the most interesting glaucoma drug carriers are either those that can be injected close to the glaucoma drug target, i.e., the aqueous humor, or solid ones able to maintain stability on the cornea, which connects them with the eye surface. Considering the excess of the drug incorporated into traditional formulations, the therapeutic effect is provided by its small fraction. It is important to be aware of this when designing novel delivery systems.

In this paper, we present the most interesting novel glaucoma drug carriers with direct contact with cornea or aqueous humor able to eliminate unfavorable drug permeation routes. In this work, we will refer to such proper delivery as a targeted delivery. We do not discuss here carriers that may promote systemic adsorption, including nanoparticles in suspensions, conjunctival or intracanalicular inserts, and subconjunctival injections [46]. We instead focus on carriers able to exclude excipients present in traditional formulations. The emphasis is put on the drug release profile, total time of release, and the therapeutic potential. If possible, the entrapped drug amount able to act therapeutically is compared to the drug amount included in corresponding traditional formulations.

### 4.2. Drug Carriers for Internal Application in Glaucoma Treatment

Direct internal administration enables direct delivery to the drug target without any loss associated with ocular barriers and permeation through unfavorable routes [47]. Although significant progress has been made in the field of implantable antiglaucoma surgical devices, or so-called microstents, there are not many literature reports on internal drug carriers associated with antiglaucoma drugs delivery.

Suprachoroidal and supraciliar space constitute the most promising target for antiglaucoma drug delivery, yet no antiglaucoma polymer carriers have been developed for this. Supraciliary delivery has only been tested for pure low-concentration antiglaucoma drug solutions, whose injection demonstrated a similar IOP-lowering effect as traditional solutions using corresponding concentrations hundreds of times higher [48]. In terms of suprachoroidal delivery, only model drug particles were studied in a two-component hydrogel/solution carrier, with good results [49].

Despite the use of intravitreal injections of pure drug solutions, there is also no research into polymeric carriers for such delivery of antiglaucoma agents. However, due to the popularity of the research into the delivery of nanocarriers to the vitreous, four types of nanoparticles were tested on rabbit model to evaluate intravitreous tissue responses, including IOP reduction [50]. Nanoparticles were made of hyaluronic acid (HA), poly (l-lactic acid) (PLLA), polystyrene (PS), and poly *N*-isopropyl acrylamide (PNIPAM). IOP reduction was significant with PLLA, PNIPAM, and PS particles, and lasted for less than three days with a mean reduction of 3, 4 and 6 (all ± 2) mmHg, respectively. The effect of HA injection was negligible. Injections had no apparent influence on the anatomical structure and thickness of retinal tissues, as well as of cornea and iris, except PNIPAM particles, which caused statistically insignificant thinning.

**Figure 1:**
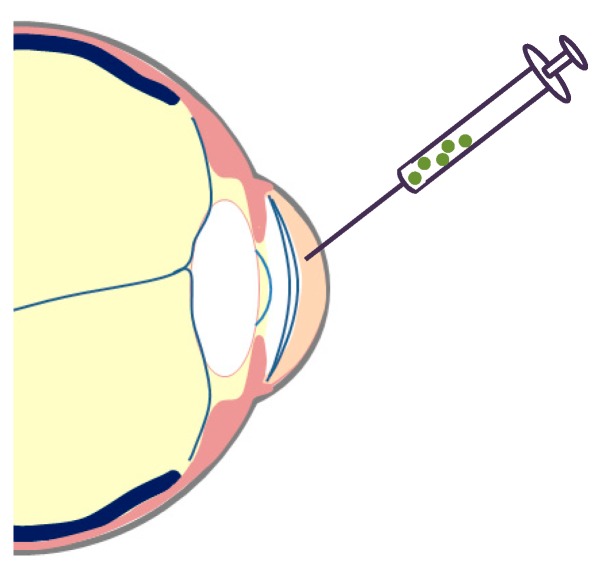
Intracameral injection scheme.

There were several attempts to form hydrogel carriers for intracameral injection (Figure 1). 

Hydrogels made of gelatin grafted with carboxylic end-capped poly(*N*-isopropylacrylamide) PN (the whole material abbreviated as GN) with pilocarpine were developed [51]. Due to the similarity to the extracellular matrix (ECM), high water content, the ability to adjust stiffness to soft tissues, and the ability to achieve the desired shape, hydrogels are a particularly interesting type of drug delivery system. In ophthalmic applications, in situ hydrogel gelation is required to allow injection in liquid form, followed by a sol–gel transition inside the human body [52].

In vitro release tests were performed in a balanced salt solution (BSS), using high-performance liquid chromatography (HPLC) for analysis. The amount of the drug incorporated in the hydrogel subjected to animal studies amounted to 1 mg. It was administered in 50 μL portion of the hydrogel solution. For comparison, the daily dosage of the drug incorporated in traditional aqueous solutions amounts to 3–4 mg.

**Figure 2:**
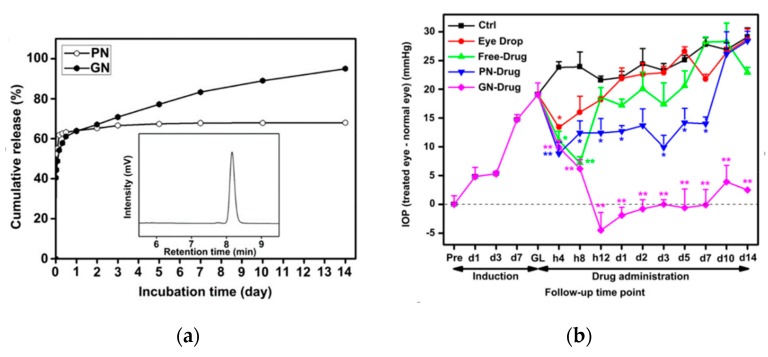
(**a**) Cumulative percent release of pilocarpine from the pilocarpine-loaded hydrogels: PNIPAAm-COOH (PN) and gelatin-g-PNIPAAm copolymer (GN); (**b**) intraocular pressure (IOP) reduction following administration of various pilocarpine delivery platforms, including gelatin-g-PNIPAAm copolymer (GN) [51].

Cytotoxicity studies revealed that the grafted hydrogel caused only little inflammation. Its movement within the intraocular space was noted. The encapsulation and in vitro cumulative release ratio were high, with the latter amounting to about 95%. Degradation of the gelatin network, surrounding the nondegradable PNIPAAm segments, was progressive, allowing for a sustained release for 14 days. After fast release of about 60% of the drug, of which the majority was released during the first 30 min (Figure 2a), it became slow and sustained. The hydrogel allowed for the several times reduction of IOP compared to traditional aqueous solution and the extension of the reduction duration, in contrast to effects obtained by other forms of drug administration (Figure 2b). Maintenance of corneal endothelial cell density after injection of copolymer hydrogel confirmed the therapeutic effect of the released drug.

Considering the small amount of the drug administered in the hydrogel and very satisfying results both in vitro and in vivo, it can be stated that the system allowed for a significant increase in drug delivery efficiency.

A polycaprolactone intracameral device loaded with a hypotensive novel agent DE-117 was developed by encapsulating DE-117 powder between thin PCL films made by spin-casting [53]. The drug amount was not given. The device was studied in vitro in terms of drug release in phosphate-buffered saline (PBS) for 10 days using high-performance liquid chromatography (HPLC) for quantitative evaluation. It was also studied on rabbit eyes in vivo in terms of IOP reduction for 24 weeks and ex vivo in terms of histology after 24 weeks and drug concentration in ocular tissues after 5, 12, and 24 weeks of implantation.

The drug was released at a rate of about 0.5 μg a day. No adverse in vivo ocular effects were observed, except one case in 16. Concentration of the drug was maintained in the aqueous humor and the target tissue up to 24 weeks. One week after implantation, IOP was reduced to about 7 mmHg from baseline, while untreated eyes experienced a reduction of 0.3 ± 2.9 mmHg, and were slightly increasing throughout the study. IOP reduction after empty device implantation was negligible compared to untreated eyes. Only the eye with ocular adverse effects exhibited histological abnormalities.

There are also reports of patent pending intracameral implants delivering antiglaucoma drugs. Biodegradable polymer implants containing 60 or 120 μg of bimatoprost in 300 or 600 μg implants, respectively, were formed [54]. The first month of drug release amounted to about 15%, giving 9 μg for a 60 μg load implant. Then, the release sped up, and after 60 days, 50 μg release was achieved. There were also presented biodegradable polymer implants containing travoprost [55]. The effect of IOP reduction on dog eyes of three simultaneously implanted materials lasted more than 7 months, and was sustained and clinically significant, varying from more than −10 to about −3 mmHg from the baseline.

In summary, five intraocular drug delivery systems were presented, including two inventions in which patents are pending. Two studies included in vivo examinations. With regard to beneficial drug pharmacokinetics, the therapy effectiveness was high, and the prolongation of IOP reduction lasted from days to several months. The release profiles of the in vitro studied systems were also promising. Although patient compliance is not high with intraocular systems, the prolonged effect they achieve prevails in their favor.

### 4.3. Drug Carriers for External Application in Glaucoma Treatment

#### 4.3.1. Drug-Loaded Contact Lenses and Contact Lenses with Drug-Loaded Coatings

There were several studies on drug release from contact lenses and contact lenses coated with drug-releasing nanofibers formed by electrospinning. Using contact lenses as drug carriers allows for maintaining a constant drug concentration on the corneal surface and, therefore, reduces the probability of systemic absorption.

Contact lenses are widely used and accepted by patients. It has also been shown that drug-eluting contacts have gained interest amongst ophthalmologists, who would prescribe them for glaucoma therapy [56]. However, the storage of therapeutic contact lenses without drug loss is challenging. At the same time, only evident oxygen permeability of contact lenses—excluding the possibility of cornea damage from hypoxia—allows for prolonged use, such as overnight wear [57].

Soaking contact lenses in drug solutions have resulted in their quick release [58]. Of the various examined methods, the incorporation of vitamin E, which acts as an obstacle to the progression of the drug, extended the release of several ophthalmic drugs [59].

Commercially available contact lenses with an oxygen permeability adequate for overnight wear were loaded with timolol and vitamin E through soaking [57]. The lenses were predesigned for multiple days’ wear. They were investigated in terms of drug release in vitro (in PBS) and IOP reduction potential in vivo on animal beagle dog model. The eyes were observed for ocular irritancy.

For in vivo studies, one set of vitamin E lenses (0.23 g) and two sets of control lenses without vitamin E were fabricated. Soaking solutions contained 200 μg for the two former sets and 60 μg for the latter. The lenses with lower drug amounts were designed to be replaced daily, and with higher amounts when worn continuously for 4 days. The exact drug and vitamin E uptake were not known. For lenses that were manufactured for in vitro drug release experiments, less-concentrated soaking solutions were used, and drug loading was estimated at about 37 μg both for lenses without vitamin E and two types of lenses with vitamin E, containing 0.09 and 0.23 g. For comparison, the daily dosage of timolol in traditional formulations amounts to 200–400 μg.

**Figure 3:**
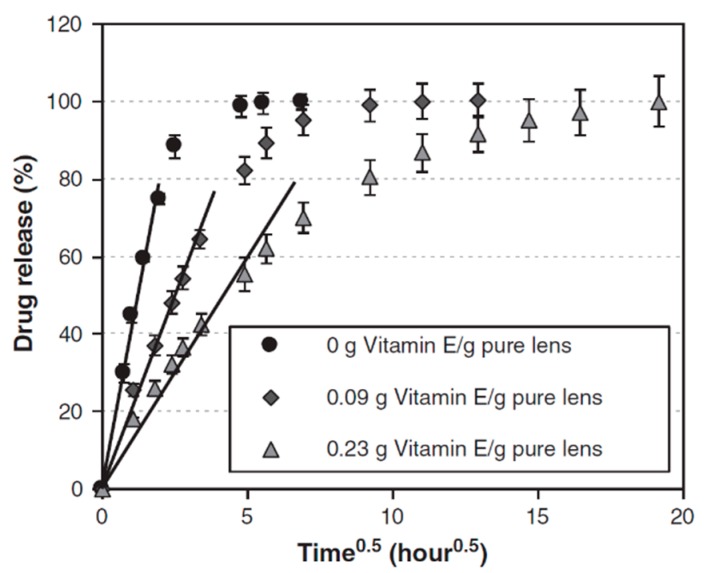
Cumulative percent release of timolol from the timolol-loaded NIGHT & DAY™ lenses with various vitamin E loadings [57].

The prolongation of the in vitro release was high in all examined lenses compared to traditional aqueous solution. In lenses without vitamin E, 80% of the drug was released during the first 4 hours and the total release time amounted to 24 hours. The drug release from contact lenses with vitamin E was more sustained—the duration of 80% release increased to 22 and 84 hours for lenses with 9% and 23% of vitamin E, and the remainder approximately 6 and 16 days, respectively (Figure 3).

**Figure 4:**
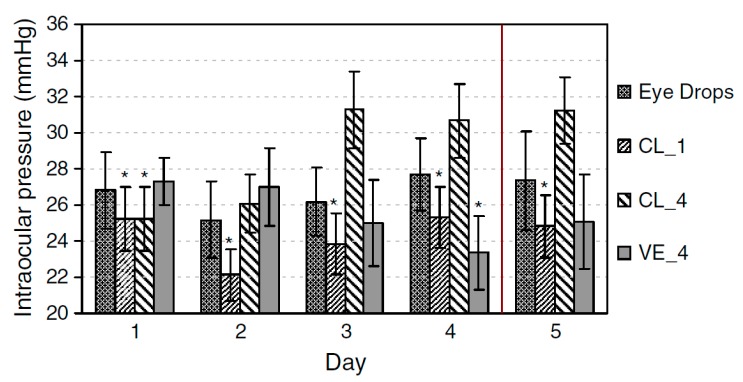
IOP reduction following administration of timolol-loaded NIGHT & DAY™ lenses with and without vitamin E [57].

Contact lenses without vitamin E with lower drug amounts allowed for the significant IOP reduction on animal model (Figure 4) of about 6 to 9 mmHg, prolonged to day 5. Lenses with higher drug amount continuously worn for 4 days were ineffective in IOP reduction. Lenses with vitamin E allowed for IOP reduction of about 4–9 mmHg, and the effect was maintained until day 5. There was also no difference in IOP in untreated eyes, confirming the lack of systemic absorption. No major discomfort, irritation, as well as other ocular adverse effects followed lens wear.

A similar approach was used for commercially available contact lenses were loaded with timolol, dorzolamide, and vitamin E [60]. Single-drug lenses and two-drug lenses were prepared by soaking lenses in PBS solutions containing timolol or/and dorzolamide. For vitamin-modified lenses, solutions were enriched with vitamin E. The drugs and vitamin E loading were estimated at 20 of timolol and 122 μg of dorzolamide in control lenses and 18 of timolol and 113 μg of dorzolamide in vitamin E lenses, with 20% of the vitamin. Drug release from single and two-drug lenses was studied (in PBS) and UV–vis spectrophotometry was used for quantitative evaluation. For both in vitro and in vivo studies, another series of lenses was prepared, containing 60 of timolol and 218 μg of dorzolamide together in control lenses and 193 of timolol and 680 μg of dorzolamide in vitamin E lenses, with 20% of the vitamin, being about ¼ of the traditional daily dose. Therapy efficacy and safety was studied in vivo on a Beagle dog model. The IOP and heart rate was measured over the 5 days of the study. Control lenses without vitamin E were worn for 24 hours, while vitamin E lenses were worn for 48 hours and then replaced. Animal eyes were observed for signs of ocular adverse effects.

**Figure 5:**
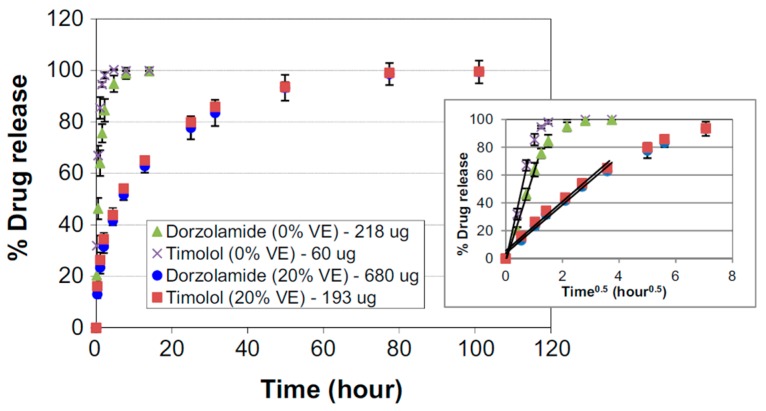
Cumulative percent release of timolol and dorzolamide released simultaneously from the same lens with or without vitamin E loaded [60].

The release durations of 90% timolol and dorzolamide lasted 42 minutes and 2.5 hours, respectively, and increased to 24.6 hours for timolol and 36 hours for dorzolamide in lenses with vitamin E. The release durations of timolol and dorzolamide from two-drug lenses achieved 1.2 and 3 hours, respectively, and significantly increased to about 42 hours for both drugs in lenses with vitamin E (Figure 5).

**Figure 6:**
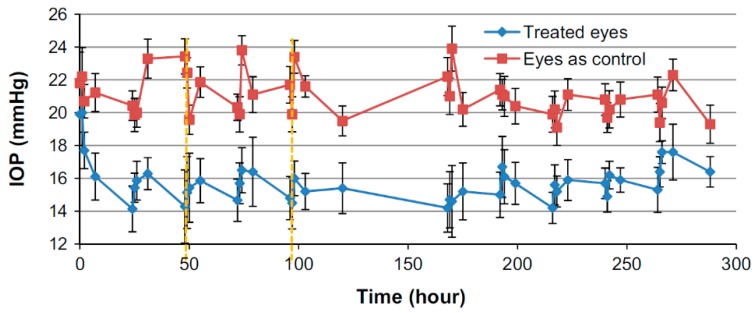
IOP reduction during and after wearing lenses with both timolol and dorzolamide with vitamin E loaded; second dash indicates lens removal [60].

IOP reduction in an animal model was significantly greater with drug-eluting contact lenses than with aqueous solution. While with aqueous solution the maximum drop of about 2.6–4.7 mmHg was observed, the IOP decrease with two-drug contact lenses over the 5 days was far more uniform and achieved 5–6 mmHg. Two-drug lenses with vitamin E allowed for an IOP reduction of 3.4–5.5 mmHg, however, the low IOP was maintained after the lens removal (Figure 6). Bearing in mind the low drug concentrations in the contact lenses, their bioavailability was significantly improved. No ocular toxicity was observed during the study.

While with the aqueous solution treatment, the average heart rate decreased 4.5% from baseline, the vitamin E lenses allowed for a significant decrease in heart rate between the first and the last 5 days, i.e., after the lenses were removed. Together with the fact that in vitamin E contact lenses therapy, the untreated eyes did not show any decrease of the IOP, while they did with aqueous solution, this could serve as a confirmation of the successful elimination of systemic absorption.

Contact lenses with latanoprost were made by encapsulating latanoprost and poly(lactic-co-glycolic acid) (PLGA) films in a typical contact lenses material by ultraviolet light polymerization [14].

Three lens types were fabricated, with two different lactide/glycolide mole ratios of PLGA—CL85:15 with an 85:15 ratio and two types of CL65:35 with 65:35 ratio. While the first amounted to 45 µm thickness, the latter two exhibited 20 and 40 µm thickness. Release studies were performed in vitro in PBS, and in vivo in the aqueous humor. Lenses were worn for 30 days. An enzyme immunoassay (EIA) kit was used for quantitative analysis of the released drug. The thicker lenses contained 4 mg of latanoprost each, and the thinner ones 2 mg.

**Figure 7:**
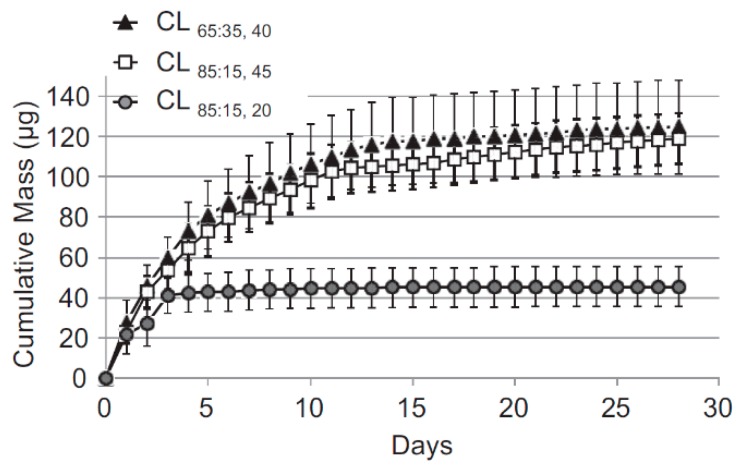
Cumulative in vitro mass release of latanoprost from latanoprost-loaded contact lenses [14].

In all cases, the release started from a burst which was followed by sustained release (Figure 7). The burst was much lower in contact lenses containing thicker drug–polymer films as the release rate following the burst was greater with them. In lenses CL65:35 (40 µm) and CL85:15, 48% and 45% of the drug was respectively released during the first 3 days, followed by a sustained 30-day release. The daily release rate with thicker films was about ten times greater than with thinner ones.

The drug concentrations in aqueous humor were comparable to average hourly concentration achieved with traditional latanoprost aqueous solution, but while with traditional solution, the reduction lasts 25 hours, the developed therapeutic contact lenses allowed for prolongation of the reduction of up to almost 30 days. Although approximately 25% of the contact lenses became displaced, the in vitro cytotoxicity studies were negative, and rabbit eyes did not develop any signs of toxicity, irritation, or other adverse ocular effects.

Latanoprost was also incorporated into silicone hydrogel contact lenses of commercial labels galyfilcon A, senofilcon A, and balafilcon A, and polyHEMA-based soft lenses of commercial label omafilcon A [61]. The drug was incorporated by 24 hours of soaking with a drug solution in PBS. Around 95% of the dissolved drug was taken up by galyfilcon A and senofilcon A hydrogels, and 98% by the balafilcon A, giving approximately 185 µg, and nearly 25% by omafilcon A, giving 50 µg per lens. The system was subjected to release studies performed differently than in other featured research using three in vitro models of cornea comprised of membranes with corneal epithelial cells. The first model consisted of a polyethylene terephthalate (PET) membrane with no cells; the second one of a PET membrane seeded with a monolayer of human corneal epithelial cells (HCECs); and the third of a PET membrane seeded with a multilayer of HCECs. The release studies were performed for 48 hours.

**Figure 8:**
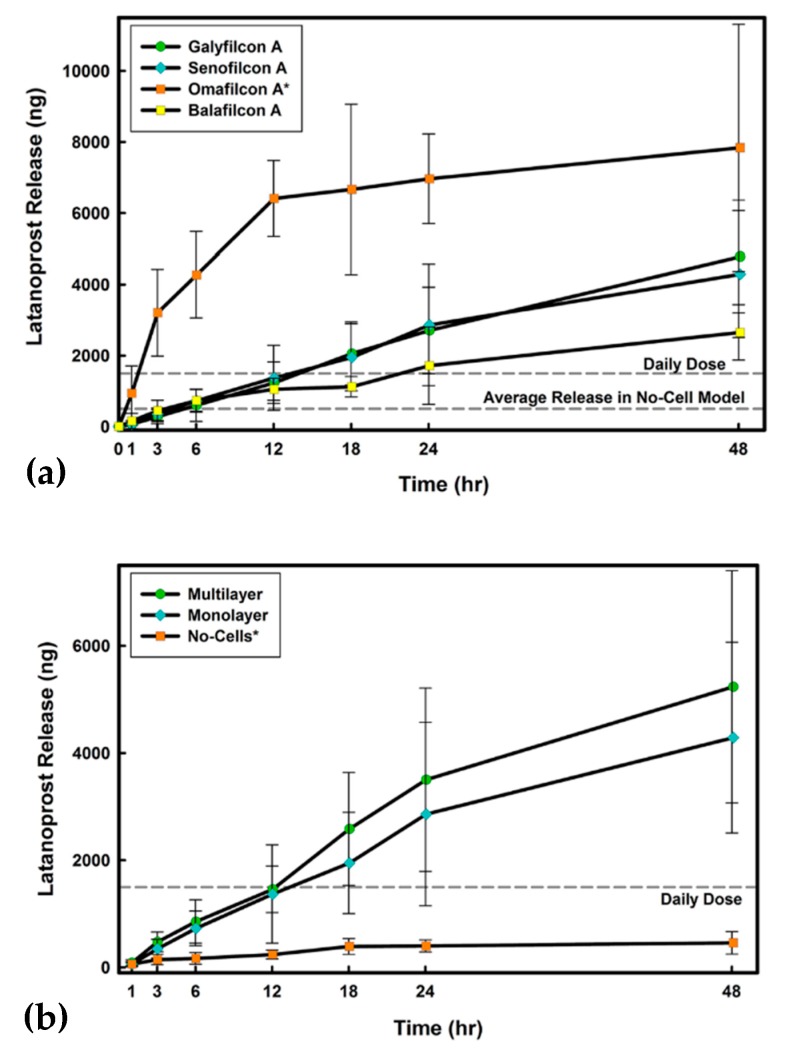
(**a**) Cumulative mass release of latanoprost from all lens types in the monolayer model; (**b**) Cumulative mass release of latanoprost from senofilcon A in the three in vitro release models [61].

In the no-cells model, an initial burst in the first 6 hours was observed with only omafilcon A. This was followed by saturation of the surrounding release medium while the drug was still available in the contact lenses. For other lens types, the burst was absent, and the release profile was almost flat. The amounts released during 48 hours amounted to tenths of a percent for silicone lenses and about 5% for nonsilicone lenses omafilcon A, with the last one achieved in the first 24 hours. In the presence of a mono- and multilayer of corneal epithelial cells, for all materials studied, a significantly higher drug release rate over time was observed (Figure 8a,b). After 24 hours, 2% of the of drug incorporated into silicone contact lenses was released. A significantly higher amount of about 10–17% in both models was released for omafilcon A. After 24 hours, the release profile did not change significantly and retained its line, except for omafilcon A, where the release rate dropped slightly.

If the release lasts longer than the experiment time, the system has enormous potential, especially for silicone lenses.

Contact lenses comprised of timolol maleate imprinted copolymer of carboxymethyl chitosan-g-hydroxy ethyl methacrylate-g-polyacrylamide (CmCS-gHEMA-g-pAAm) embedded onto a polyHEMA matrix (pHEMA) were formed [62]. They were intended for reloading, while most of previously developed imprinted lenses were disposable. In vitro release studies were performed in a solution imitating the lacrimal fluid. The carrier was loaded with 20 µg of the drug. The single drug dose amounts to 100–200 µg, which doubles in daily dose; however, the authors determined it to be 1.7 µg. After each cycle of drug release, contact lenses were immersed in normal saline to remove unreleased drug and reloading was done in drug solution in water.

**Figure 9:**
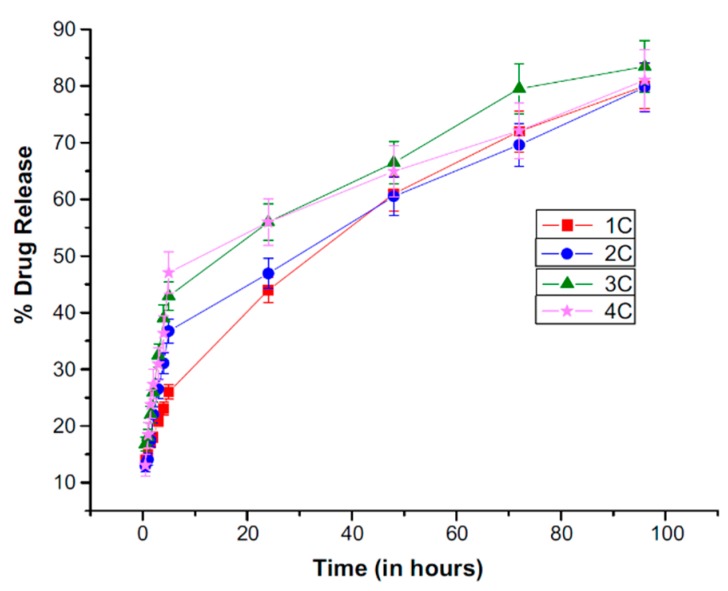
Cumulative percent release of timolol maleate from timolol-loaded contact lenses after first, second, third, and fourth cycle of drug loading [62].

The encapsulation rate after the first loading achieved 85.5%, 72%after the second, and decreased with every subsequent cycle. The drug release following each reloading was sustained and lasted about 90 hours (Figure 9). Considering the amounts incorporated and amounts contained in traditional formulations, the delivery effectiveness was much increased, however, considering the amount estimated by the authors, the amount incorporated in the carrier extended the needs.

The mechanical stability was described as proper for the extended wear. The maintenance of timolol maleate activity following each drug reloading and release was decreased only slightly in comparison with pristine drug. The cytotoxicity of the material was negligible.

Timolol maleate loaded nanofibers made of PVP and poly(*N*-isopropylacrylamide) in 1:1 ratio (PNIPAM) containing 5% or 15% of timolol maleate (% *w*/*w* of polymer weight) with eight various permeation enhancers (one for one formulation) were electrospun on soft contact lenses [63]. They were predestined to be worn throughout the day and removed at night. However, the lenses used as precursors are required to be stored in the aqueous medium, which would significantly speed up the release, if not completely clear out the drug from the nanofibers. This application aspect was not described in the publication. Still, they present a promising drug delivery system. In vitro drug release tests were performed in PBS via cellophane dialysis membrane. The coating was electrospun from solution containing about 12.5 or 37.5 mg of the drug. As a reminder, the daily dosage contained in traditional formulations amounts to around 200–400 μg.

**Figure 10:**
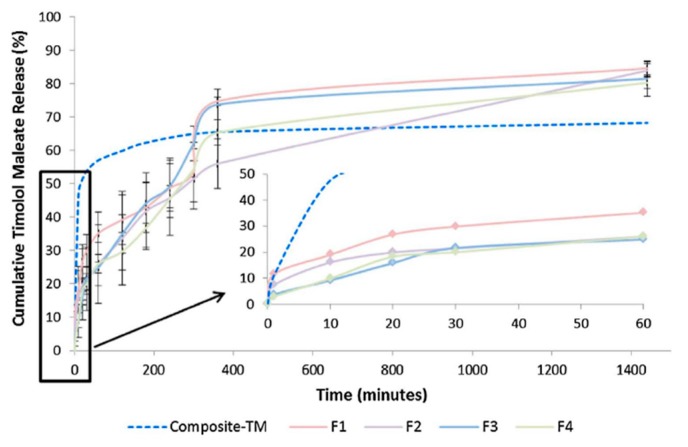
Cumulative percent release of timolol maleate from timolol-loaded (5% *w*/*w*) contact lenses with various permeation enhancers [63].

Timolol maleate release varied depending on the permeation enhancer located in the nanofibers. In all cases, the release profile was changing in time, after about 6 hours of fast release of linear or quasi-linear profile slowing down to achieve slower, sustained release (Figure 10). Burst was observed with some nanofibers, the smallest with those containing EDTA with 15% of timolol, Brij 78 with 5% timolol, and borneol both with 5% and 15% timolol. Moreover, borneol addition allowed for obtaining the most sustained drug release and the amount of timolol released was increased by 20%, compared to permeation enhancer-free nanofibers.

As a result of the intended time of application, the release was measured for only 24 hours. This time allowed for the release of about 80% of the entrapped timolol in all cases. Considering traditional daily dose, it exceeded the needs. The Bovine Cornea Opacity and Permeability (BCOP) test revealed no damage to the cornea when exposed to contact lenses containing all types of permeation enhancers and higher polymer content (15%), proving the material’s biocompatibility.

In conclusion, six studies on the therapeutic contact lenses were presented, of which five included in vivo studies on animals. Three systems were designed to be worn continuously for 48 hours, 4 days, and 30 days. In two other studies, either the lenses were reloaded daily or the problem of lens storage was neglected. However, the application time did not affect ocular tissues and no ocular adverse effects were observed in any case. The IOP reduction was maintained throughout the duration of each study and in two cases it was prolonged after carriers’ removal. The release profiles of in vitro studied system were also promising.

#### 4.3.2. Nanofibers

Electrospun nanofibers are well known for allowing sustained and controlled delivery of many active compounds. It is the result of their high surface to volume ratio as well as porosity of both nonwoven and single nanofibers. With proper selection of materials and process parameters, electrospun nanofibers have the ability to trap a large amount of particular drug, maintaining the chemical activity and stability of its structure [64].

Majority of biodegradable polymeric materials can be formed into fibers, including pure polymers, their blends, copolymers or dendrimers. While the first three are enriched with drugs at the stage of solution preparation, dendrimers are functionalized with drugs, which become part of their structure [65].

There are three main mechanisms of drug release from nanofibers: desorption of the drug located on the nanofiber surface, diffusion of the drug located into the nanofibers and drug release associated with the polymer matrix degradation (erosion). The parameters controlling the release are related to the nanofiber properties and properties of the polymer which form them. The rapid initial release called burst release, which is often observed, is attributed to the fraction of the drug, which is adsorbed or weakly bound to the large surface area of polymer nanofibers, rather than to the drug incorporated in polymer nanofibers [64].

Nanofibrous materials in the form of sheets or smaller patches can be easily placed close to the drug target as in the case of therapeutic contact lenses. However, they have a significant advantage of not covering the whole conjunctiva, allowing air and nutrients penetrate to the eye surface. They should possess mucoadhesive properties, as adhesion to the cornea is not ensured by other features, such as lens’ cornea-like curvature. The nanofibrous structure is light and, with sufficient mucoadhesion, can persist on the cornea for the total time of the application. When biodegradable at an adequate rate, it does not have to be removed, even after the time of drug release and delivery. In conclusion, this allow for the local maintenance of therapeutic effects over a long time, reduction of the drug dose, increase of the effectiveness and safety of the therapy and, finally, for the increase of the patients’ compliance.

Nanofibers made of modified polyamidoamine (PAMAM) dendrimers and poly(ethylene oxide) containing brimonidine tartrate (BT/DNF) have been investigated [66]. The nanofiber carrier was predestined to be put on the cornea daily and dissolve rapidly. The drug release kinetics in vitro was performed in simulated tear fluid (STF) via cellulose dialysis membranes and the absorbance of the drug in dialysates was measured. Drug permeability ex vivo across rabbit corneas was investigated using Franz diffusion cells and reverse phase HPLC equipped with a UV detector was used for a quantitative evaluation. The nanofiber patch for in vivo study contained 40 μg of the drug. For short-term study, the single-dose response was measured. For long-term study, the patch was administered daily for 21 days. The single dose in traditional aqueous solution amounts of 70 μg and 140 μg of the drug is in a daily dose.

**Figure 11:**
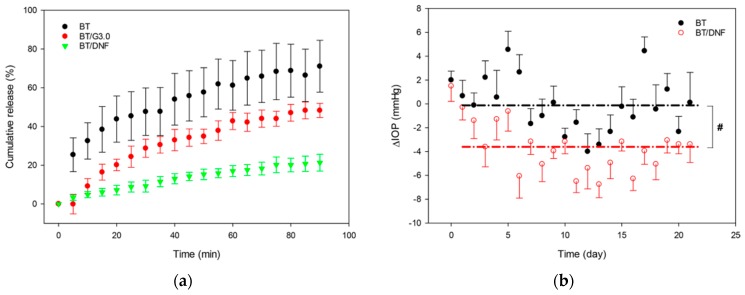
(**a**) The cumulative percent release of brimonidine from brimonidine-loaded nanofibers of G3.0-mPEG and poly(ethylene oxide) (BT/DNF) compared to release from brimonidine aqueous solution (BT) and PAMAM dendrimers (BT/G3.0); (**b**) IOP reduction following administration of brimonidine-loaded nanofibers (BT/DNF) compared to the IOP-reducing potency of brimonidine aqueous solution (BT) [66].

The drug release was measured for only 90 min and the release did not achieve even 20% of the administered dose. The drug release profile was flattened and there was no burst (Figure 11a). Nanofiber patch administered to rabbit eyes dissolved instantly. The drug efficacy after single-dose administration was equivalent between nanofibers and traditional aqueous solution. In an in vivo 3-week study of daily application, the IOP presented a downward trend throughout the study, achieving significantly lower average values compared to those obtained with aqueous solution (Figure 11b).

The discussed nanofibers caused no toxicity in vitro, and neither ocular irritation or signs of inflammation were observed in animals in the single-dose response tests. During the chronic use trial, only redness of the eye surrounding skin was observed. Considering the drug dosage, the obtained efficacy of the system was very high. However, it is a question to be asked whether such a high dissolving rate is beneficial for the release and delivery. The daily patch replacement seems to squander the potential of a longer mucoadhesive nanofibers application, as well as the potential of increasing patient compliance.

Other nanofibrous patches consisted of poly(vinyl alcohol) PVA, poly(ε-caprolactone) PCL, and a combination of timolol maleate and dorzolamide as active compounds [67]. It was predestined by the authors to be put in the conjunctival sac to limit its contact with cornea. The authors justified their choice of destination place by the fact that conjunctival sac can retain the maximum volume of the administered traditional aqueous solution. Although it is a fact, this way of permeation is definitely not the most proper for the therapeutic effect. The presented patch was, however, attractive and could be applied on the corneal surface, which could enhance its therapeutic effect. In vitro drug release in artificial tear fluid via activated cellophane membrane was studied, and the quantitative analysis was performed with the use of UV spectroscopy. The drug entrapment efficiency amounted to about 100%, but no reference values were given, so it was impossible to make a conclusion about the drug delivery efficiency compared to traditional timolol/dorzolamide formulations.

**Figure 12:**
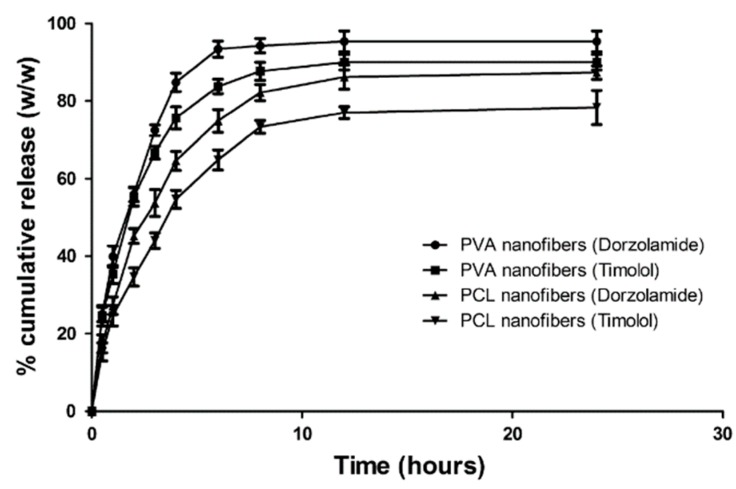
Cumulative percent release of timolol maleate and dorzolamide from timolol/dorzolamide-loaded PVA and PCL nanofibers [67].

In vitro release studies showed that the patches were capable of controlled drug delivery up to 24 hours, although almost 100% of the drug was released in the first 9 hours (Figure 12). The mucoadhesive strength of the system was described as enabling placement in the eyes for a longer period.

**Figure 13:**
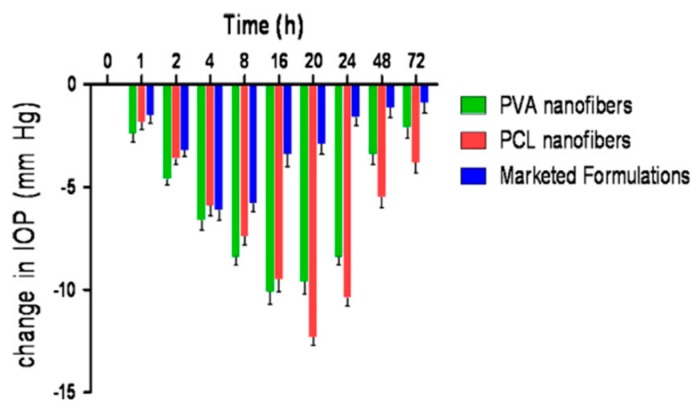
IOP reduction following administration of timolol/dorzolamide-loaded PVA and PCL nanofibers compared to the IOP-reducing potency of traditional (marketed) formulations [67].

The reduction in IOP was major compared to traditional formulation (Figure 13). While the maximum reduction with traditional formulation was achieved 4 hours after administration, the nanofibrous patch allowed this time to be enhanced to 20 hours, and after 72 hours, the achieved reduction was 4 times greater. PVA nanofibers did not cause any eye irritation while PCL nanofibers caused minor irritations.

In conclusion, two studies on therapeutic nonwovens were presented, both formed using the electrospinning technique. One of them was designed to be applied on the cornea daily and dissolve rapidly while the second one was to be put in the conjunctival sac for an unknown period. Only the former was studied using animal eyes (ex vivo). Although the presented results were satisfying, either the long-term potential was not exploited or location of the carrier was inappropriate, which could be visible in a long-term study. The fast dissolving system could be examined in terms of IOP reduction after its removal. To the best knowledge of authors, no other papers on nanofibrous patches for glaucoma applications have been published yet and there is still potential for nanofibers in ophthalmic applications to be used in the future.

## 5. Conclusions

In this paper, we discussed novel glaucoma drug carriers whose planned location place—and, thus, drug delivery—was narrowed down to the closest area of glaucoma drug targets. Most achieved a high and prolonged therapeutic effect, in most cases, much higher than that obtained with corresponding traditional glaucoma formulations, and in some cases, this was even maintained after carrier removal. This was shown either by the potential of IOP reduction or the drug concentration in aqueous humor. In all cases, smaller or much smaller drug doses were used compared to traditional formulations, showing a significant improvement in bioavailability. In some cases, a lack of systemic absorption was demonstrated. Most of the research highlighted the systems’ capability to reduce disadvantages coming from traditional formulations and confirmed this by accurate observations and instrumental studies on animal models. We believe that the work clearly showed the huge benefits of novel antiglaucoma drug delivery systems and the potential they bear.

It was however noted that values of IOP described as being normal and the reference value in in vivo tests varied between articles. As it impedes reliable assessment and can be misleading when conducting a comparison of systems, we believe that they should always correspond with the IOP values presented by the healthy eye of an animal model. The medium used in in vitro release studies was also not unified. PBS, being the most common choice, significantly differs in composition from normal human tear film, while there are other salts or buffers that are more suitable (e.g., Tyrode’s salts, BSS ophthalmic irrigation solution).

Within the reviewed systems, the injectable carriers displayed the most beneficial results in terms of prolongation of the therapeutic effect. However, because they require a medical procedure, attention is directed to therapeutic contact lenses. They have much higher patient acceptance, while achieving beneficial effects even up to 30 days. The effect of an IOP reduction after their removal, observed in two cases, would allow for making pauses in the application. From the presented literature, we are convinced that despite few successes, electrospun nanofibers have strong potential for application as an antiglaucoma drug delivery system. Studies on nanofibrous drug delivery systems for various other purposes show that they can effectively deliver molecules in a controlled manner for a long period of time [64]. Although elderly patients might have difficulties with accepting both contact lens and nanofibrous carriers, we believe that many of them will adapt, especially since less frequent application will be required. An additional advantage is the lack of head tilting, difficult with dizziness, degenerative cervical spine, vertebrobasilar circulatory disorders, and many other conditions.

The delivery platforms in existence are refined, but much is to be done in the future. It is essential to support each research with beneficial results by in vivo studies. In our opinion, it is important to design systems with the full use of their potential in terms of drug delivery duration together with encapsulation rate. Hopefully, glaucoma drug delivery provided by means of traditional formulations will soon be replaced or, at least, largely supported by novel targeted glaucoma systems.
